# Analysis and consensus of currently available intrinsic protein disorder annotation sources in the MobiDB database

**DOI:** 10.1186/1471-2105-14-S7-S3

**Published:** 2013-04-22

**Authors:** Tomás Di Domenico, Ian Walsh, Silvio CE Tosatto

**Affiliations:** 1 Department of Biology, University of Padova, Viale G. Colombo 3, 35131 Padova, Italy

## Abstract

**Background:**

Intrinsic protein disorder is becoming an increasingly important topic in protein science. During the last few years, intrinsically disordered proteins (IDPs) have been shown to play a role in many important biological processes, e.g. protein signalling and regulation. This has sparked a need to better understand and characterize different types of IDPs, their functions and roles. Our recently published database, MobiDB, provides a centralized resource for accessing and analysing intrinsic protein disorder annotations.

**Results:**

Here, we present a thorough description and analysis of the data made available by MobiDB, providing descriptive statistics on the various available annotation sources. Version 1.2.1 of the database contains annotations for ca. 4,500,000 UniProt sequences, covering all eukaryotic proteomes. In addition, we describe a novel consensus annotation calculation and its related weighting scheme. The comparison between disorder information sources highlights how the MobiDB consensus captures the main features of intrinsic disorder and correlates well with manually curated datasets. Finally, we demonstrate the annotation of 13 eukaryotic model organisms through MobiDB's datasets, and of an example protein through the interactive user interface.

**Conclusions:**

MobiDB is a central resource for intrinsic disorder research, containing both experimental data and predictions. In the future it will be expanded to include additional information for all known proteins.

## Background

Intrinsic protein disorder is becoming an increasingly important topic in protein science [1-3]. Protein function has been traditionally thought to be determined by tertiary structure. Over the last decade, intrinsically disordered proteins (IDPs) have been found to be important in many important biological processes [4-6]. IDPs are widespread in natural proteins, especially in eukaryotic organisms [7,8], and are frequently associated with molecular recognition [9,10]. They have been observed to be common among hub proteins, i.e. those with many interaction partners [11] and also to play a key role in human disease [12]. In addition, protein disorder is important for experimental protein characterization since difficulties often arise when long disordered regions are present, which frequently happens at the N and C termini [13]. IDPs represent a heterogeneous concept with many different and elusive definitions [14] which can be traced back to different indirect experimental methods.

### Sources of disorder information

Currently available sources for intrinsic disorder annotations can be divided in two main groups. The first group includes annotations inferred from experiment, with evidence in publications. The second group includes annotations automatically extracted by computational tools. The latter can be further subdivided into automatic annotations derived from experimental sources, and automatic annotations obtained from software predictors.

There are currently two available sources of intrinsic protein disorder information with evidence in publications. The DisProt [15] database, a manually curated repository, features disorder and structure annotations for 667 proteins (version 6.00). The IDEAL [16] database, also manually curated, contains information on 209 proteins. The Protein Data Bank (PDB) [17] constitutes the main source of available experimentally-based disorder annotations with over 70,000 different structures. It is widely accepted that missing residues from X-ray structures have a good correlation with intrinsically disordered residues [18]. These missing regions can easily be extracted from structure files deposited in the PDB. Some 6,000 structures solved by NMR experiments are generally deposited as structural ensembles in a single file. These can be used to detect residue mobility [19] which, in a way that is analogous to the missing X-ray regions, are a good indicator of intrinsic disorder. NMR structures were only recently considered in disorder prediction [20], demonstrating the long held belief of different flavours of disorder [1,3,21].

A great number of intrinsic disorder predictors have been developed over the last few years [22], with two main scenarios emerging for their application. The first is represented by predictions of disorder on a relatively small number of proteins with maximum accuracy, such as in the CASP experiment [23]. Most existing prediction methods, such as Disopred [7], VSL1 [24] and CSpritz [25], have been trained for this scenario. A more practical scenario is however represented by the genome-scale analysis of disorder [1,8], where some performance is sacrificed to achieve results in a reasonable time frame. This usually entails using a method that does not require a multiple-sequence alignment, thereby speeding up computation by several orders of magnitude [20]. DisEMBL [26], IUPred [27] and, more recently, ESpritz [20] have been all developed with this scenario in mind.

In the following, we will describe the construction of the MobiDB database of experimental and predicted disorder annotations in proteins [28]. In particular, we will compare the different annotation sources and how they are integrated. A coherent consensus disorder definition will be derived and used to annotate the proteomes of a set of representative model organisms.

## Materials and methods

### Database structure

**Figure 1:**
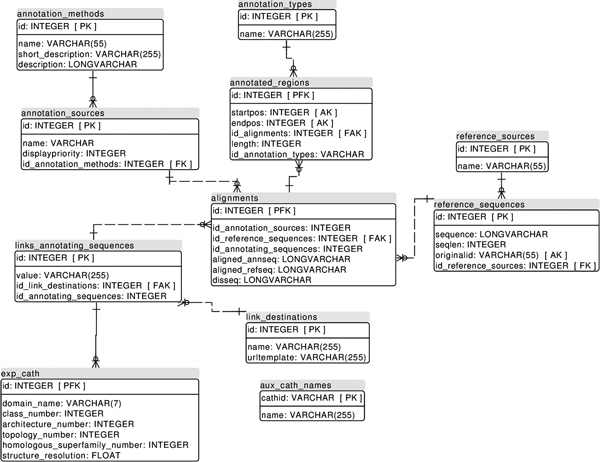
**Database schema for MobiDB**.

MobiDB [28] data is stored and queried using the PostgreSQL database engine. The database schema is composed of 11 tables and shown in Figure 1. The main idea in the database is to have a set of reference protein sequences, which will be annotated by associating as many annotating sequences as possible to them. The reference sequences represent distinct biological objects, e.g. proteins, which can be obtained with unique identifiers from a reference collection such as UniProt. Annotating sequences are obtained from the various sources mentioned in the previous section. They can be mapped at residue-level to the reference sequences, and provide information such as e.g. disorder, secondary structure, and sequence conservation. In principle, the database schema can be used for any sequence-based annotation. The data is partially normalized, although some exceptions to the normal forms have been introduced with the aim of improving efficiency when inserting and querying data.

Data loading is performed as a three-step process. In the first step, annotations are extracted from each annotation source and stored as two Fasta files. One of these files contains the annotating sequences, and the other the annotations extracted from those. An extra comma-separated file is generated which links the annotating sequences to their corresponding reference sequences. In the second step, a script takes the first step output files and generates tab-separated files compatible with the database engine's batch-loading mechanism. During this step, if an annotating sequence covers only part of its corresponding reference sequence, an alignment between the two is performed. The potential resulting gaps introduced in the annotating sequence are also transferred to the extracted annotation. The third and final step consists simply of loading the data in batch to the database. To maximize the loading performance, the affected database indices are dropped before the insertion begins. The resulting database constitutes the backend of the application, which will then be accessed by the user interface.

The middle tier of MobiDB is composed of Java Servlets. These receive a query from the front-end, submit it to the backend, and translate the results into hierarchical Java objects. These objects are then transformed into JSON objects, and made available for further processing by the front-end. MobiDB's user interface makes extensive use of modern internet browser features to provide a flexible user experience. The results provided by the middle-tier are processed and displayed using the JQuery JavaScript library. A widget system was developed which allows for the display of information in independent UI subunits that can be rearranged throughout the screen by the user to fit its needs.

### Disorder data and resources in MobiDB

All of the aforementioned disorder sources are integrated into the MobiDB database. XML files from the DisProt and IDEAL databases are parsed for annotations. Information on the corresponding UniProt entries to be linked to those sources is included in also included in the XML files. Annotating sequences from PDB files are extracted by means of custom scripts (X-ray) and the MOBI server (NMR). These annotations are then linked to their corresponding UniProt protein sequences by means of the SIFTS database [29]. In order to capture different flavours of disorder, seven *in silico *disorder predictors are run against all the reference sequences: Three Espritz [20] flavours (X-ray, NMR, DisProt) and two flavours each for IUpred [27] (short, long) and DisEMBL [26] (remark465 and hot loops).

MobiDB version 1.2.1 integrates the latest versions of its data sources at the time of writing. It features a total of 4,662,776 proteins and covers all complete proteomes for eukaryotic species, as present in the UniProt database [30]. Table 1 provides a detailed list of such sources and their corresponding versions.

**Table 1:** **Overview of the databases used in ModiDB 1.2.1**. The databases used and relevant references are listed with the description of extracted information and the version or download date included in MobiDB.

Database	Reference	Information extracted	Version/Date
UniProt	[30]	Reference sequences	2012-07

DisProt	[15]	Disorder and structure	6.00

IDEAL	[16]	Disorder and structure	2012-05-09

PDB	[17]	Disorder and structure	2012-08-15

SIFTS	[29]	UniProt-PDB links	2012-08-15

Pfam	[34]	Functional domain annotations	Web service

OMA Browser	[31]	Protein orthologs	2012-03

CATH	[35]	Structural classification	3.4

DSSP	[36]	Secondary structure	2012-08-15

### Disorder consensus and weighting

For each protein with experimental annotations, the average of annotating sequences in the MobiDB database is seven. The annotations from *in silico *predictors are excluded from this average, since they require only the protein sequence as input and can therefore provide annotations for all proteins in the database. Furthermore, different disorder annotation sources may reflect different types of disorder phenomena. Given these facts, a simple method to combine annotations would allow for a more integral vision of disorder information. With this in mind, we developed a novel consensus disorder annotation that integrates all available disorder annotations for a protein. The consensus is calculated for each position of the reference sequence, by taking into account the corresponding positions in the annotating sequences whenever they are available. It is composed of two values: *disorder level*, and *annotation score*. The disorder level evidences how much the selected annotations agree on whether a given position of the reference sequence is structured or disordered. It is an integer value ranging from 0 to 9, with 0 meaning full agreement on a region being structured, and 9 meaning full agreement on a structure being disordered. It is calculated by the following formula:

(1) $$l=\text{min}\;\left(round\left(\frac{10*\sum dw}{\sum dw+\sum sw}\right),9\right)$$

where Σdw is the sum of weights of annotations considering the region disordered, and Σsw is the sum of weights of annotations considering the region structured. The annotation score evidences the strength of a given consensus annotation. It is the sum of the weights of every annotation that agrees with the final consensus for a certain region. Its objective is to allow the classification of regions according to the amount of data backing up the resulting annotation. This amount is also dependent on the relative weight of each annotation. In all cases, the sums are calculated over all the annotations corresponding to a certain position of the reference sequence. This may be visualized as the columns in an alignment between the reference sequence and its corresponding annotating sequences. In the case where an annotating sequence has no annotation for a certain reference sequence position, its contribution to the sum is zero. In all cases the minimum value of the sums is zero, and the maximum will depend on the number of annotations available, and the weight assigned to each of them.

Empirical weight factors have been derived for each disorder annotation source. Intuitively, the rationale is to favour manually curated annotations (DisProt and IDEAL) over experimental structures from the PDB, and the latter over all predictors. The weighting factors were thus chosen to resemble this situation, with X-ray structures judged depending on resolution and preferred over NMR models. The weights for annotations obtained from the DisProt and IDEAL databases were chosen so that having a few high resolution X-ray structures can tilt the disorder consensus towards ambiguity as these are may represent regions of alternating structure. DisProt and IDEAL annotations are assigned a weight of 3, to reflect the quality of the manually curated data. Each X-ray annotation is given a weight according to the following formula, which increases the weight as the resolution of the experiment improves:

(2) $$W_{xray}=1-\frac{\text{log}\left(r\right)}{\text{log}\left(rT\right)}$$

where *r *is the resolution of the experiment, and *rT *is a user-defined maximum resolution threshold. This threshold allows the user to set a baseline in the form of a minimum resolution required for a structure to provide a significant annotation. In the case where the resulting weight is smaller than 0.2, a fixed value of 0.2 is assigned. PDB NMR structures are assigned a fixed weight of 0.2 each, to reflect the usually higher uncertainty in coordinates obtained by NMR experiments when compared to their X-ray counterparts. Finally, predictor-generated annotations are given a weight of 0.05, which allows experimentally obtained data to prevail whenever it is available.

### Sequence conservation and disorder classification

In order to provide information regarding the sequence conservation of disorder, MobiDB [28] also annotates sequence conservation on groups of orthologous protein sequences. For each reference sequence in the database, a search is performed in the OMA Browser database [31] to look for a corresponding group of orthologs. If such a group is found and contains at least 10 members, a multiple sequence alignment is constructed with CLUSTALW [32]. A position in the alignment is considered conserved if the same residue is present in at least 50% of the sequences. Whenever such sequence conservation annotations are available, disordered regions in reference sequences are classified in a way analogous to the definitions introduced by Bellay and co-workers [33]. If the region is disordered and its sequence conserved, it is defined as "constrained disorder". If, on the other hand, the region is disordered but the sequence not conserved, it is termed "flexible disorder".

## Results and discussion

In order to assess the available information on disorder, it was first necessary to create a new database. MobiDB was thus designed with three main goals in mind: performance, scalability and usability. The database had to maintain good performance both when loading, so it can be updated frequently, and querying, so as to be useful for the public by providing fast response times. It had to be scalable, meaning that performance levels can be maintained when expanding with further information. Last but not least, it had to provide high levels of usability, giving the user a centralized, flexible and useful way to access intrinsic disorder information in an intuitive way. Updates for MobiDB are carried out through a three-step loading process integrated into a single, automated pipeline (see Methods). This allows for the easy regeneration of the entire database with up-to-date information in less than a week's time. Enabled by this fact, and based on the update frequencies of the different sources integrated into MobiDB, we have set a quarterly update interval. Every three months MobiDB will be updated to keep up with recent additions to its information sources.

### Use cases for MobiDB

There are two main use cases for MobiDB. The first one is the analysis of a single protein by means of the user interface. The second one is the generation of a custom dataset for offline analysis. Both actions are available after performing a database search, or after accessing one of the browse options. MobiDB supports the UniProt complex search syntax, through a web service call to the UniProt server. This allows to build sophisticated queries with various filters, e.g. organisms and subcellular localizations. All proteins matching the search parameters will be listed along with relevant information for each entry in the *Search results *page.

From the search results, the user can click on a protein name and be directed to the *Protein *analysis page. This page features four interactive widgets, each containing different pieces of information regarding the selected protein. The *Reference sequence information *widget contains general information related to the chosen reference protein, extracted from the UniProt database. The *Annotation sources *widget contains the different annotated regions from each annotating sequence that has been linked to the reference sequence. The *Annotations plot *widget provides a graphical representation of the available annotations associated to the reference sequence. This contains general annotations such as Pfam annotations and disorder consensus, as well as all available disorder annotations sources.

Instead of analysing a single protein via the graphical interface, the user can opt to download a dataset containing multiple entries. This can be done by pressing the download button in the top left of the search results page. The exported dataset will is composed of two fasta files. One of them containing all relevant reference and annotating sequences and the other one containing all the corresponding annotations. Pre-computed datasets are available in the download section of the MobiDB website for the different experimental data sources, as well as for each of the 297 complete proteomes.

### Analysis

Given the unifying concept of MobiDB, where different disorder data sources are collected and serve to annotate the same sequences, it is interesting to note how these sources relate to each other. In an effort to quantify the differences and similarities and to allow for the comparison, Table 2 provides a variety of descriptive statistics. The left half of the table contains residue-level information, while the right half contains region-level information. We define a disordered region as a consecutive stretch of residues annotated as disordered. The residue-level data gives a quick picture on the amount of information each source contributes. It also shows how generous each of them tends to be when annotating a residue as disordered or structured. The region-level data evidences the length distribution of the regions detected by each source. As can be expected, the different disorder sources contain data with different characteristics. There appears to be two well-defined clusters and some outliers. The PDB-xray, ESpritz-xray and IDEAL annotations appear to concentrate on few residues with somewhat longer disordered regions. This can be rationalized as sequence segments which probably do not crystallize. DisProt tends to annotate regions of similar length to the previously mentioned sources, but mostly contains only disorder annotations, yielding more disordered residues. On the other hand, the PDB-nmr, ESpritz-nmr, DisEMBL-hl and, to a lesser degree, the IUPred sources tend to annotate a larger amount of residues, but grouped in shorter regions. This likely can be explained as flexible regions fluctuating in space, which may or may not be entirely disordered. ESpritz-disprot is an outlier which predicts comparatively few residues as disordered, but when disorder is predicted it is for very long segments.

**Table 2:** **Comparison between disorder data sources**. The different disorder data sources are compared in terms of available sequence entries and distribution of ordered and disordered residues. The distribution of disordered regions is also shown in terms of the lowest (1^st^) and highest (3^rd^) quartiles, median and mean.

		Residues			Disordered region lengths			
**Source**	**Entries**	**Annotated**	**Disordered**	**Fraction disordered**	**1^st ^quartile**	**Median**	**Mean**	**3^rd ^quartile**

**DisProt**	794	84,671	79,820	0.943	8	20	58.88	63

**IDEAL**	207	47,967	6,077	0.127	5	16	49.95	62.25

**PDB-nmr**	7,556	642,252	120,117	0.187	4	7	19.72	22

**PDB-xray**	180,373	47,309,921	2,400,507	0.050	5	20	88.96	132

**DisEMBL-465**	4,662,776	2,070,982,327	238,367,624	0.115	13	26	81.34	85

**DisEMBL-HL**	4,662,776	2,070,982,327	529,251,895	0.255	12	20	41.92	45

**ESpritz-disprot**	4,662,776	2,070,982,327	165,087,066	0.080	18	102	239.1	347

**ESpritz-nmr**	4,662,776	2,070,982,327	621,164,099	0.300	6	14	34.71	36

**ESpritz-xray**	4,662,776	2,070,982,327	252,651,437	0.122	6	25	106.1	128

**IUPred-long**	4,662,776	2,070,982,327	462,197,994	0.223	1	4	28.97	22

**IUPred-short**	4,662,776	2,070,982,327	404,986,684	0.195	2	5	32.69	25

**Figure 2:**
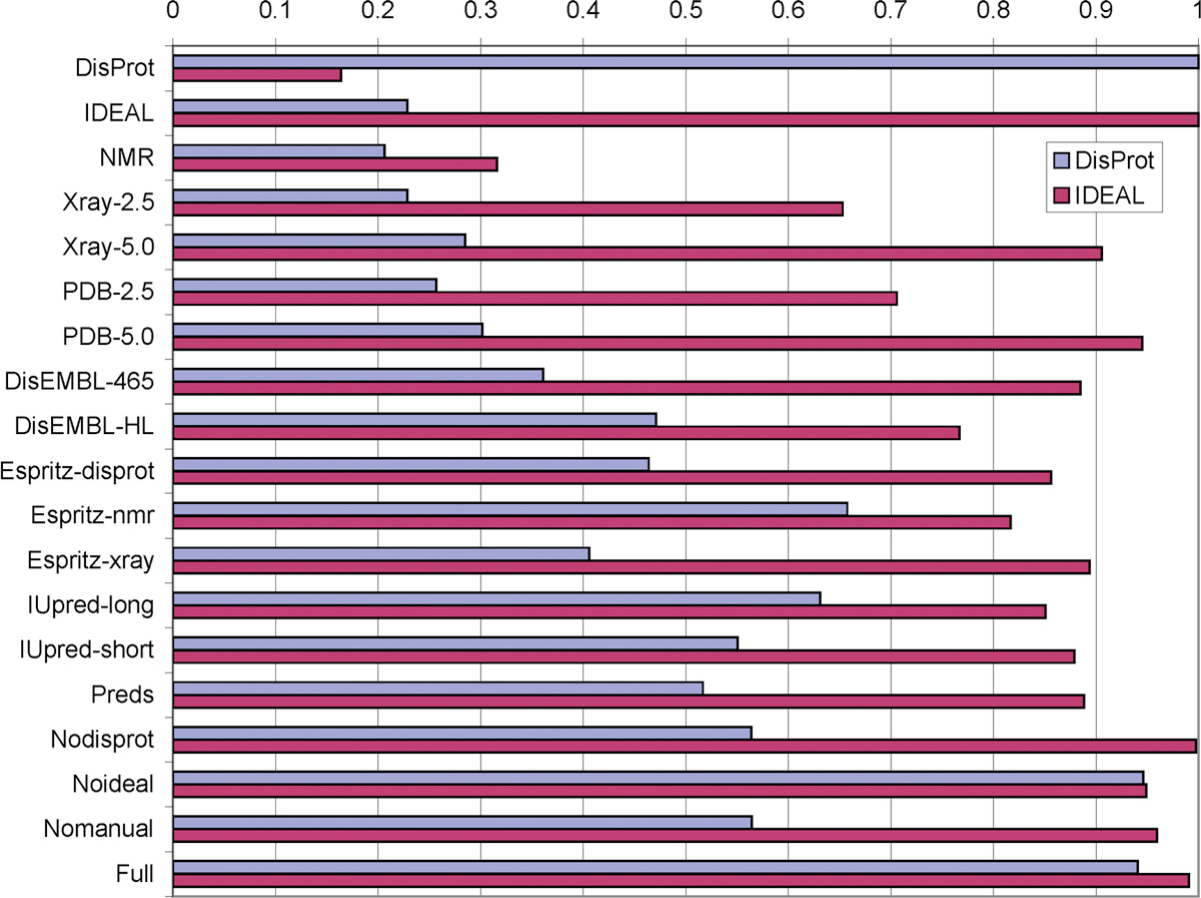
**Agreement of disorder sources and consensus with the DisProt and IDEAL annotations**. Results are shown as agreement of each data source with the DisProt and IDEAL reference datasets. Notice the difference between DisProt and IDEAL, and how the latter is mainly similar to PDB information. Overall, it is interesting to see that IDEAL is much easier to replicate than DisProt, suggesting a relative lack of long disordered regions in the former.

A second test was carried out to better understand the relationship between the different disorder data sources, as defined in Table 3, and manually curated disorder definitions. Figure 2 shows the agreement between each source and the DisProt and IDEAL annotations used as gold standard. Here, matches or mismatches are only considered when a curated annotation exists. The first striking result is that the two gold standards, DisProt and IDEAL, are rather different. In fact, for proteins with both annotations, the reproducibility of one from the other is around 20%. This is rather puzzling, given how both strive to describe the same phenomenon. Upon closer inspection, it becomes apparent that IDEAL focuses more on shorter disordered regions, which more readily correspond to missing X-ray residues. DisProt on the other hand contains more longer disorder segments. In general, it is harder to reproduce the DisProt annotation than IDEAL.

**Table 3:** **Overview of the disorder definitions used**. The labels used for disorder data sources throughout the paper are defined. The type column lists whether the source contains experimental information (Exp), predictions (Pred) or consensus (Cons).

Label	Definition	Type
DisProt	DisProt database annotations	Exp

IDEAL	IDEAL database annotations	Exp

NMR	PDB NMR annotations	Exp

Xray-2.5	PDB Xray annotations, resolution threshold of 2,5 Å	Exp

Xray-5.0	PDB Xray annotations, resolution threshold of 5 Å	Exp

PDB-2.5	PDB-xray and PDB-nmr annotations, resolution threshold of 2,5 Å	Cons, Exp

PDB-5.0	PDB-xray and PDB-nmr annotations, resolution threshold of 5 Å	Cons, Exp

DisEMBL-465	DisEmbl remark 465 predictions	Pred

DisEMBL-HL	DisEmbl hot loops predictions	Pred

Espritz-disprot	ESpritz DisProt predictions	Pred

Espritz-nmr	Espritz NMR predictions	Pred

Espritz-xray	Espritz XRay predictions	Pred

IUpred-long	IUPred long predictions	Pred

IUpred-short	IUPred short predictions	Pred

Preds	All predictors	Cons, Pred

Nodisprot	Full MobiDB consensus without DisProt	Cons, Exp, Pred

Noideal	Full MobiDB consensus without IDEAL	Cons, Exp, Pred

Nomanual	Full MobiDB consensus without manually curated data (DisProt and IDEAL)	Cons, Exp, Pred

Full	Full MobiDB consensus (all sources)	Cons, Exp, Pred

### Consensus

**Figure 3:**
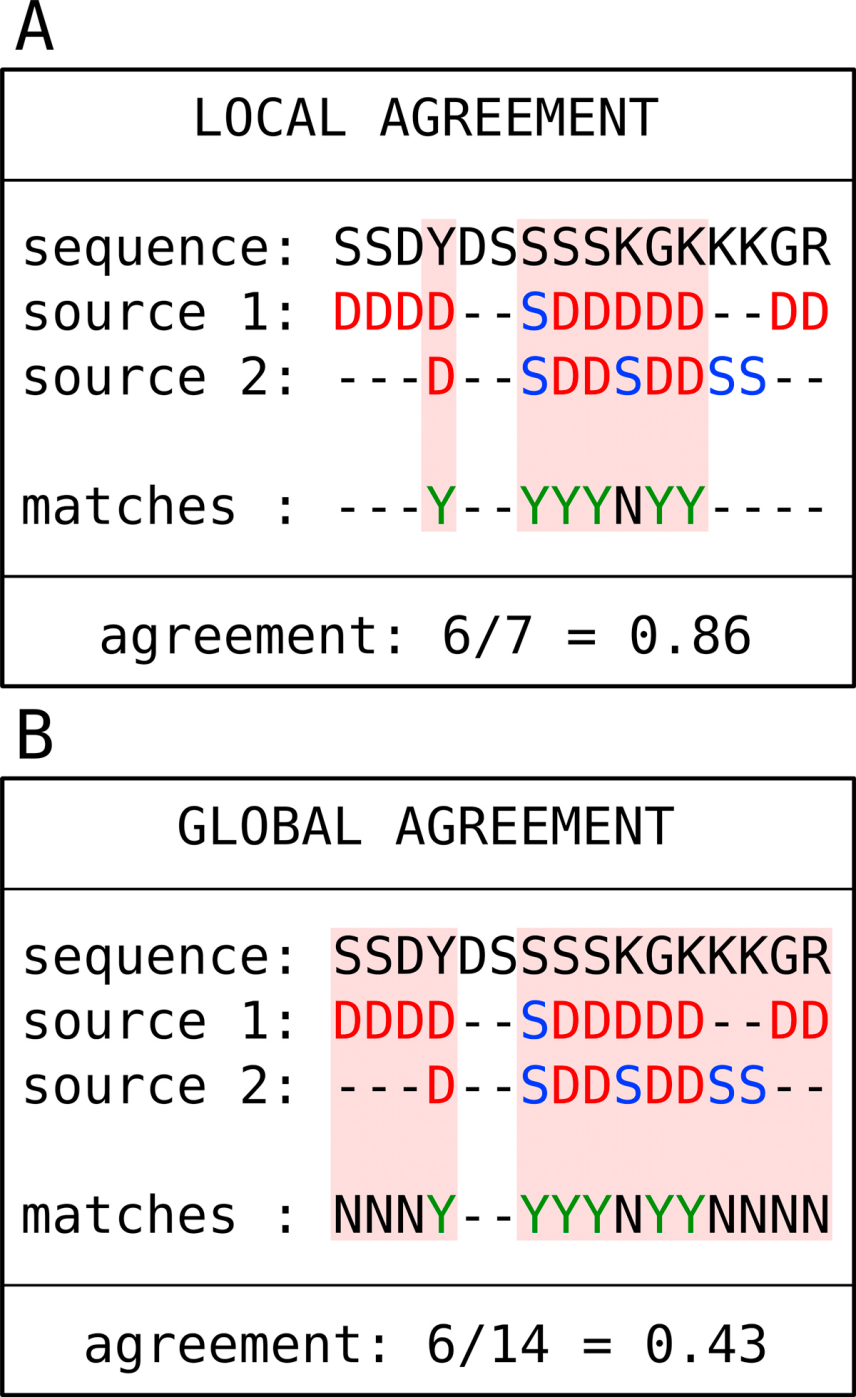
**Agreement definitions**. Schematic representation of two alternative agreement definitions between two disorder annotation sources mapping to the same sequence stretch. In the source lines, D is used for disorder and S for structured. The match line shows the agreement between sources with Y (yes) used for agreement and N (no) for disagreement.

**Figure 4:**
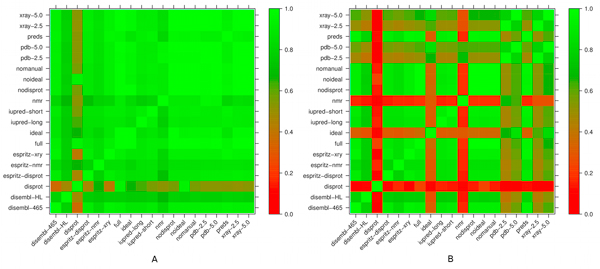
***Agreement *among disorder sources**. The agreement among pairs of disorder data sources is plotted from red (0.0) to green (1.0) using the two definitions local (A) and global (B) agreement from Figure 3. Even though the different sources have a high level of local agreement, the number of times two sources annotate at the same time is relatively low. The global agreement evidences this by showing a drastic drop in agreement when the situation of one of the sources annotating, and the other not, is considered a negative. The MobiDB consensus aims at combining different sources to maximize the coverage when annotating a reference sequence, trying to overcome this issue.

From the various disorder data sources it is a logical step to derive a consensus annotation. The protocol for this is described in Methods and a set of variants defined in Table 3 are also tested in Figure 2. The predictor consensus agrees ca. 50% of the time with DisProt, but covers almost 90% of the IDEAL annotations, again reinforcing the impression about the differences between these two gold standards. In general, the full consensus was designed to closely reproduce the manually curated data whenever available. This analysis can be taken one level further, by showing the level of agreement between each possible combination of annotations, by building "restricted" consensus annotations that include only a subset of the disorder information sources. Three different ways to calculate the agreement are defined in Figure 3, defining whether gaps in one annotation should be considered or not. Figure 4 shows the results for these definitions, which are broadly similar with the baseline agreement (Figure 4c) being perhaps the most representative. Most sources are rather similar, with the notable exceptions of DisProt, PDB-nmr and, to a lesser degree, IDEAL. The former two have a low agreement with the other sources and among themselves, reinforcing the notion of their unique contribution to disorder. IDEAL confirms its rather good agreement with PDB-xray data.

### Proteome analysis

**Figure 5:**
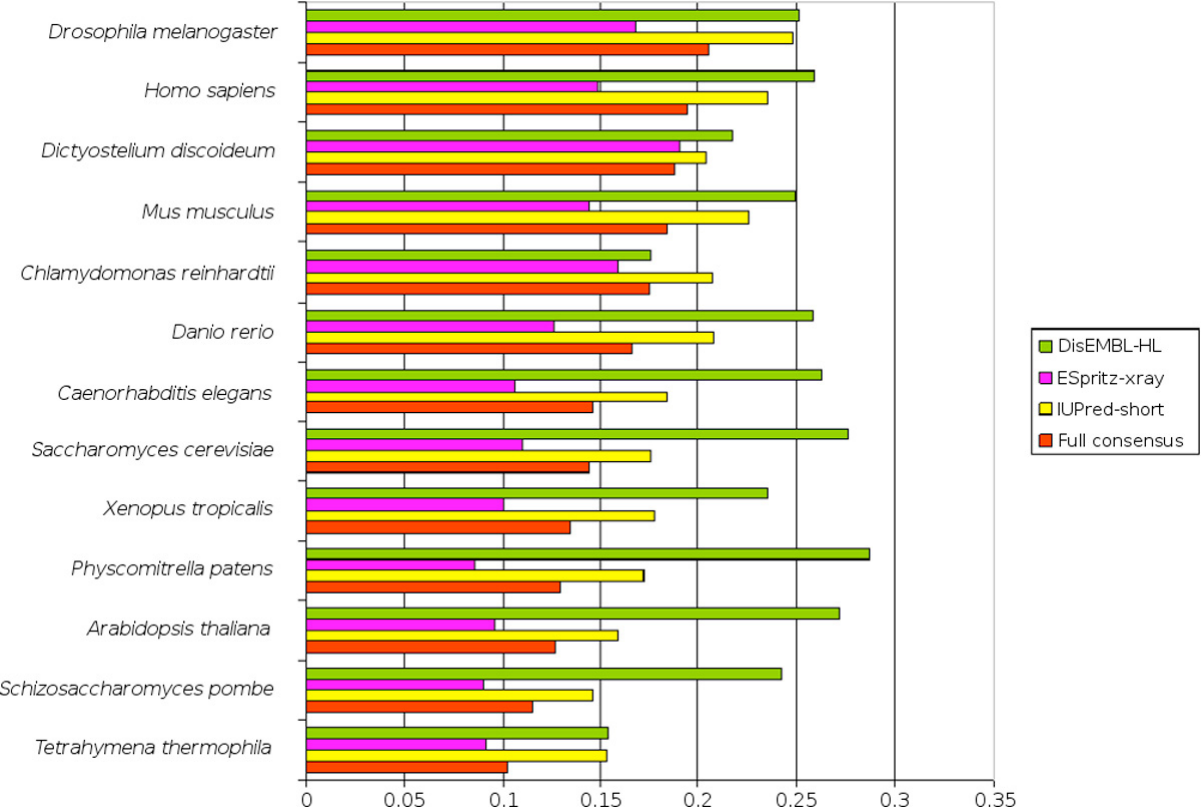
**Percentage of disordered residues on all proteins encoded by a selection of model organisms**. The fraction of disordered residues is recorded for a group of model organisms according to the full MobiDB consensus and three chosen predictors. Data is and sorted increasingly according to MobiDB consensus. Notice how DisEMBL-HL is the only predictor to break the broad trend for more disorder in higher organisms. The effect is however smoothed by the MobiDB full consensus. See main text for an explanation.

As an example of the potential of MobiDB, we present an analysis of disorder in 13 eukaryotic model organisms (see Figure 5). Our analysis is in broad agreement with previous data suggesting a correlation between organism complexity and disorder [7]. The overall fraction of disordered residues is lower than in previous publications, with an average or only 15% for the disorder consensus. Due to different disorder sources covering slightly different sequence stretches, effectively cancelling out each other, this estimate should be considered a lower bound only. Somewhat surprisingly, a few simple organisms are predicted to have more disordered residues than more complex ones (Figure 5). A similar observation was recently made for a larger set of eukaryotic proteomes, leading the authors to speculate about an organism's lifestyle [8]. In any case, MobiDB provides the means necessary to easily carry out proteome-wide comparisons of disorder distributions.

### Single protein analysis

**Figure 6:**
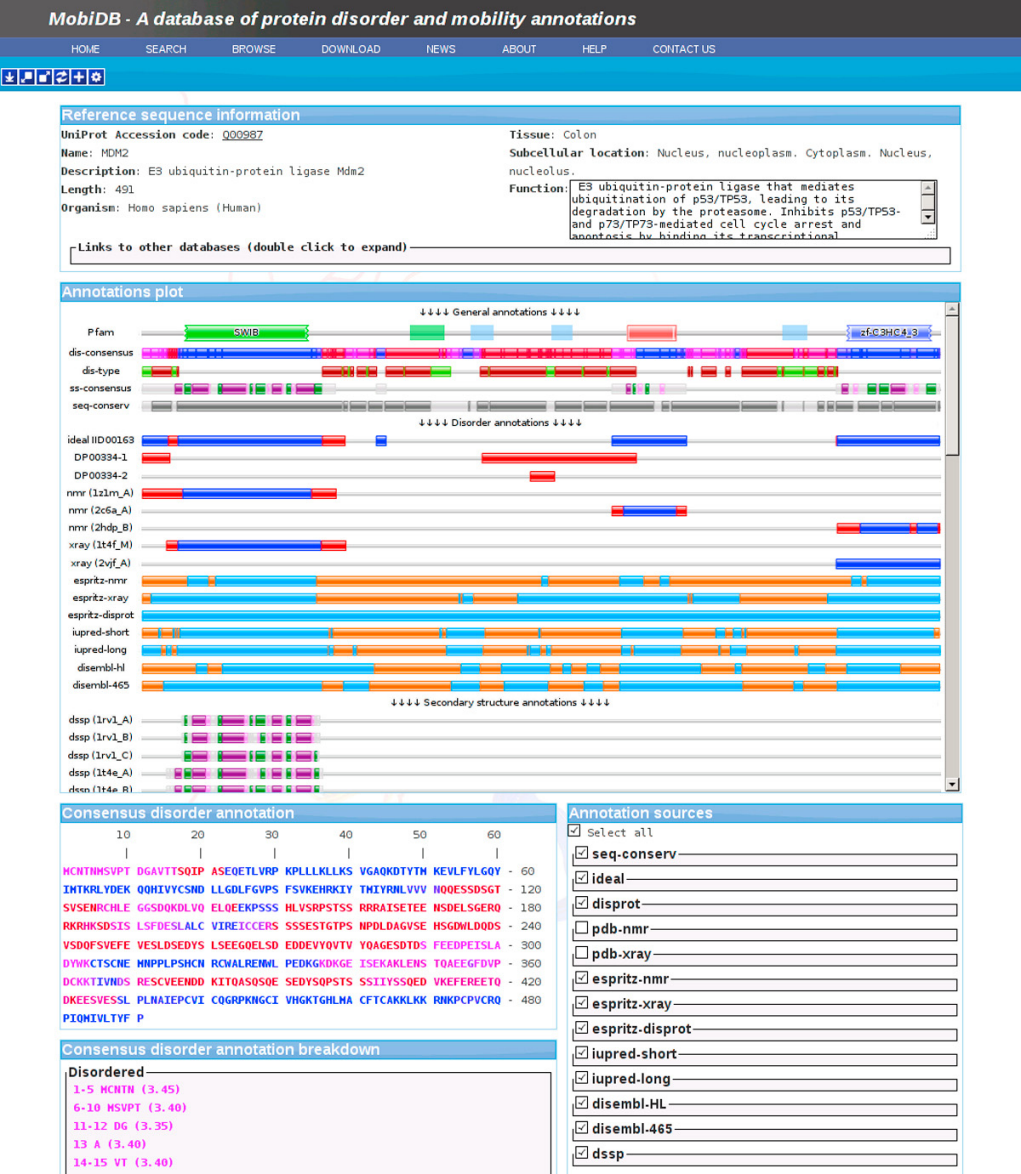
**The E3 ubiquitin-protein ligase Mdm2 in the MobiDB interactive user interface**. The interactive user interaface of MobiDB allows the user to build a customized consensus based on annotations of interest. The example shows how the database facilitates the easy integration of different data sources to maximize coverage of disorder annotations. In the example, annotations extracted from the IDEAL and DisProt databases and from X-ray and NMR experiments of the PDB are complemented by predictions to provide accurate annotations covering the full extent of the protein's sequence.

For the use case of analysing a single protein MobiDB provides an interactive user interface. In this interface the user can customize the resulting consensus by selecting only those sources of information relevant to the analysis being performed. In the case of the *E3 ubiquitin-protein ligase Mdm2 *(Figure 6), experimental annotations are available from the IDEAL and DisProt databases, and from PDB X-Ray and NMR experiments. None of these, however, provide coverage for the full protein sequence. The MobiDB consensus provides the means to elegantly combine the available annotations, allowing the user to quickly understand how disorder is distributed in his protein of interest.

## Conclusions

We have presented a detailed description of MobiDB, a database of experimental and predicted disorder in proteins, and its main features, disorder consensus and weighting. The database is highly modular and extensible, allowing inclusion of a growing amount of information. A comparison between different disorder data sources highlights how the MobiDB consensus captures the main features of intrinsic disorder and correlates well with the manually curated datasets from DisProt and IDEAL. In more detail, the DisProt curation is best approximated with a combination of disorder predictors, allowing a robust estimation of the presence of disorder in eukaryotic genomes, roughly confirming the higher incidence of disorder in higher organisms. In the future we plan to expand MobiDB to include additional information for all known proteins, both from experimental sources and new predictors, with the goal of making it an increasingly useful, centralized source of data for intrinsic disorder research.

## Competing interests

The authors declare that they have no competing interests.

## Authors' contributions

TDD and SCET designed the study, analyzed the results and wrote the paper. TDD and IW performed the experiments. All authors have read and approved the final manuscript.

## Declarations

This project was supported by funding from the University of Padova (CPDA098382, CPDR097328), FIRB Futuro in Ricerca (RBFR08ZSXY) and Cariplo (2017/0724) to S.T.

This article has been published as part of *BMC Bioinformatics *Volume 14 Supplement 7, 2013: Italian Society of Bioinformatics (BITS): Annual Meeting 2012. The full contents of the supplement are available online at http://www.biomedcentral.com/bmcbioinformatics/supplements/14/S7
